# Differential Proteomics Identifies Reticulocalbin-3 as a Novel Negative Mediator of Collagen Production in Human Cardiac Fibroblasts

**DOI:** 10.1038/s41598-017-12305-7

**Published:** 2017-09-22

**Authors:** Ernesto Martínez-Martínez, Jaime Ibarrola, Amaya Fernández-Celis, Enrique Santamaria, Joaquín Fernández-Irigoyen, Patrick Rossignol, Frederic Jaisser, Natalia López-Andrés

**Affiliations:** 1 Cardiovascular Translational Research. Navarrabiomed (Fundación Miguel Servet), Instituto de Investigación Sanitaria de Navarra (IdiSNA), Pamplona, Spain; 2 INSERM UMRS 1138 Team 1, Centre de Recherche des Cordeliers, University Pierre and Marie Curie, Paris, France; 3 Proteored-ISCIII, Proteomics Unit, Navarrabiomed, Departamento de Salud, Universidad Pública de Navarra, IDISNA, Navarra Institute for Health Research, Pamplona, Spain; 4 INSERM, Centre d’Investigations Cliniques- Plurithématique 1433, UMR 1116 Université de Lorraine, CHRU de Nancy, FCRIN INI-CRCT, France

## Abstract

Cardiac fibrosis is characterized by an excessive accumulation of extracellular matrix components, including collagens. Galectin-3 (Gal-3) and Cardiotrophin-1 (CT-1) are two profibrotic molecules that mediate Aldosterone (Aldo)-induced cardiac fibrosis. However the underlying mechanisms are not well defined. Our aim is to characterize changes in the proteome of human cardiac fibroblasts treated with Aldo, Gal-3 or CT-1 to identify new common proteins that might be new therapeutic targets in cardiac fibrosis. Using a quantitative proteomic approach in human cardiac fibroblasts, our results show that Aldo, Gal-3 and CT-1 modified the expression of 30, 17 and 89 proteins respectively, being common the reticulocalbin (RCN) family members. RCN-3 down-regulation triggered by Aldo, Gal-3 and CT-1 was verified. Treatment with recombinant RCN-3 decreased collagens expression in human cardiac fibroblasts through Akt phosphorylation. Interestingly, CRISPR/Cas9-mediated activation of RCN-3 decreased collagen production in human cardiac fibroblasts. In addition, recombinant RCN-3 blocked the profibrotic effects of Aldo, Gal-3 and CT-1. Interestingly, RCN-3 blunted the increase in collagens expression induced by other profibrotic stimuli, angiotensin II, in human cardiac fibroblasts. Our results suggest that RCN-3 emerges as a new potential negative regulator of collagen production and could represent a therapeutic target in the context of cardiac fibrosis.

## Introduction

Cardiac fibrosis is a global health problem associated with heart diseases, characterized by net accumulation of extracellular matrix (ECM) components^1^. Fibrosis disrupts the coordination of myocardial excitation–contraction coupling in both systole and diastole and may result in the development of heart failure (HF)^2^. The cardiac fibroblast is an essential cell type within the myocardium that provides structural support maintaining ECM integrity^3^. In addition, they are also essential for other physiological functions that are determined by dynamic and coordinated cell-cell and cell-matrix interactions. Its function is therefore beyond being a simple regulator of ECM production^4^. Upon injury, cardiac fibroblasts transform to a myofibroblast phenotype and contribute to cardiac fibrosis^3^. Understanding the cardiac fibroblasts and the molecular mechanisms involved in the development of cardiac fibrosis could help to the development of potential therapies that effectively target this cell type and its pathological contribution to disease progression.

Aldosterone (Aldo) acts classically by binding to mineralocorticoid receptor (MR) and is a key regulator of blood pressure and electrolytic balance^5^. Previously, we demonstrated that Aldo increased collagen type I protein levels in human cardiac fibroblasts^6^. In previous studies from our group we have identified galectin-3 (Gal-3) and cardiotrophin-1 (CT-1) as two molecules up-regulated by Aldo/MR^5–7^. Gal-3 is a β-galactoside–binding lectin family^8^ expressed in several cells including cardiac fibroblasts^9^. Gal-3 directly increases the synthesis of ECM components and mediates the profibrotic actions of Aldo in cardiac fibroblasts^6^. CT-1 is a member of the interleukin-6 superfamily produced by cardiac fibroblasts^10^ that exerts fibroblast proliferation and collagen production^11,12^. Importantly, Gal-3 and CT-1 emerge as key factors in the cardiac remodeling induced by Aldo associated with cardiac hypertrophy and fibrosis, facilitating cardiac dysfunction^6,13^.

Despite the cardiac profibrotic actions of Aldo, Gal-3 and CT-1, the underlying mechanisms are not well defined. In order to identify the common molecules or pathways activated by Aldo, Gal-3 and CT-1 in cardiac fibroblasts leading to collagen production, a proteomic approach was performed. We identified Reticulocalbin 3 (RCN-3) as a common molecule down-regulated by Aldo, Gal-3 and CT-1. Moreover, RCN-3 emerges as a new potential negative regulator of collagen production and could represent a promising therapeutic target in the context of cardiac fibrosis.

## Results

### Proteome-wide exploration of Aldo, Gal-3 and CT-1 effects on adult human cardiac fibroblasts

In order to obtain a deep insight into the protein content and protein function modulated by Aldo, Gal-3 and CT-1 on cardiac fibroblasts, a proteome-wide analysis of total cell extracts was performed using isobaric tags (iTRAQ) coupled to 2D nano-liquid chromatography tandem mass spectrometry. Using this workflow, changes of expression of 30 Aldo-modulated proteins were observed after 24 h of treatment (6 up- and 24-down-regulated proteins). Gal-3 modulated protein expression for 17 proteins after 24 h of treatment (2 up- and 15-down-regulated proteins) and CT-1 altered protein expression of 89 proteins after 24 h of treatment (54 up- and 35-down-regulated proteins).

Interestingly, Gal-3 induced a reduction in reticulocalbin (RCN)-1 and RCN-3 protein expressions, while CT-1 treatment decreased RCN-2 protein levels (Supplementary Table 1). These data suggest that RCN family could be a potential target of both profibrotic molecules.

### Effects of Aldo, Gal-3 and CT-1 on RCN family in human cardiac fibroblasts

**Figure 1:**
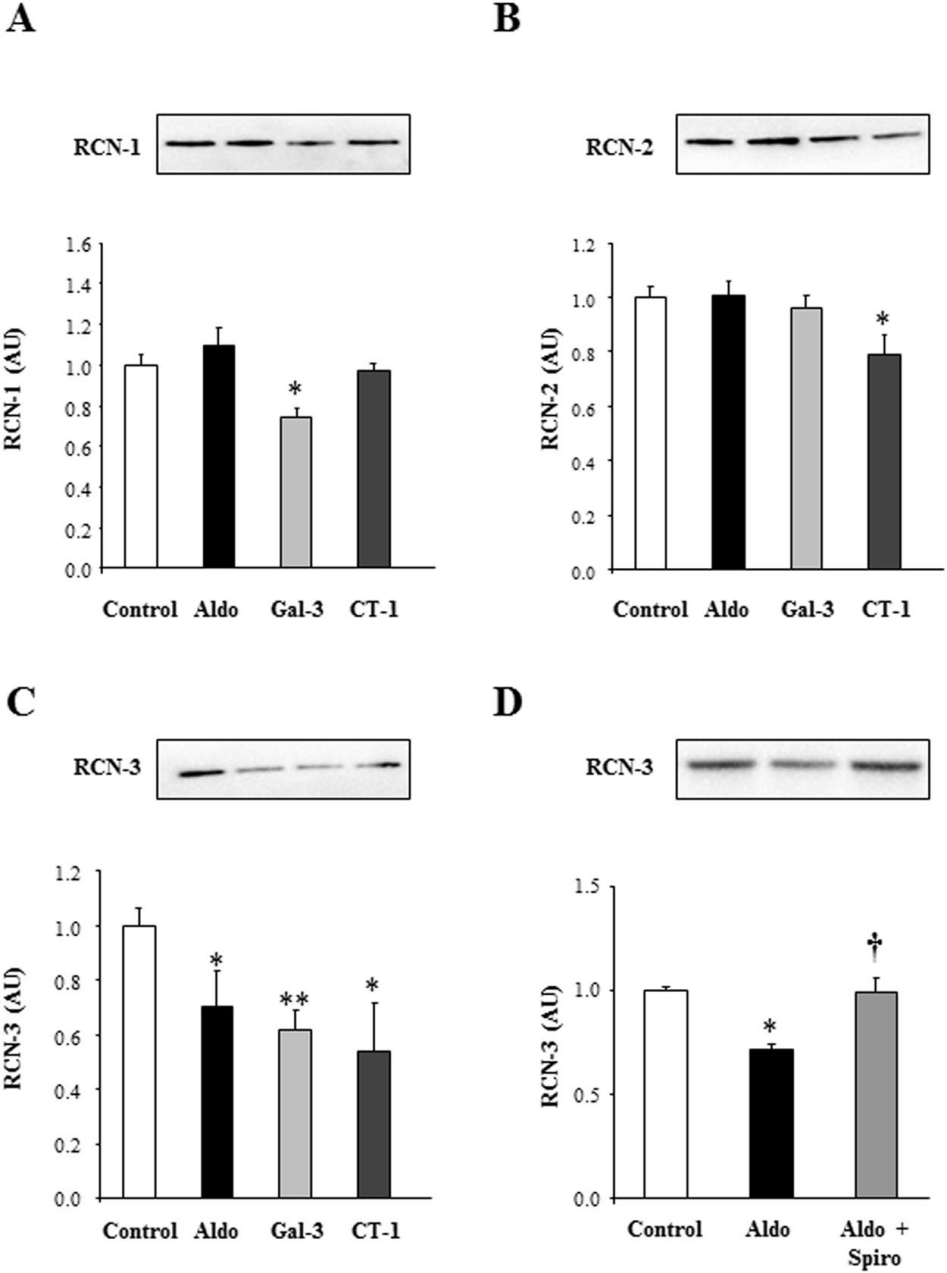
Effects of Aldo, Gal-3 and CT-1 on RCN family in human cardiac fibroblasts. Effects of Aldo (10^−8^M), Gal-3 (10^−8^M) and CT-1 (10^−7^M) on RCN-1 **(A)**, RCN-2 **(B)** and RCN-3 **(C)** in human cardiac fibroblasts. Effects of Spiro (10^−6^M) on RCN-3 protein levels induced by Aldo in human cardiac fibroblasts **(D)**. Histogram bars represent the mean ± SEM (n > 6 wells at 70% of confluence per condition) of the three independent experiments. Densitometry values were normalized to stain free gel. *p < 0.05; **p < 0.01 *vs*. Control. ^†^p < 0.05 *vs*. Aldo.

With the aim to complement and validate quantitative proteome measurements, subsequent experiments were performed in order to check the steady-state levels of a subset of differential proteins using downstream assays. In particular, we studied the effects of Aldo, Gal-3 and CT-1 on RCN-1, RCN-2 and RCN-3 in human cardiac fibroblasts. Aldo was able to decrease RCN-3 protein levels without any modification in RCN-1 or RCN-2 levels (Fig. 1A–C). Gal-3 decreased RCN-1 and RCN-3 without modification in RCN-2 levels (Fig. 1A–C). CT-1 decreased RCN-2 and RCN-3 protein levels, but it did not modify RCN-1 expression (Fig. 1A–C).

RCN-3 mRNA levels were not modified under any of the treatments at 24 hours (Supplementary Figure S1).

The presence of Spirolonactone, a MR antagonist, was able to block the decrease in RCN-3 protein levels induced by Aldo in human cardiac fibroblasts (Fig. 1D).

### Protein interactome network modulated by RCNs

**Figure 2:**
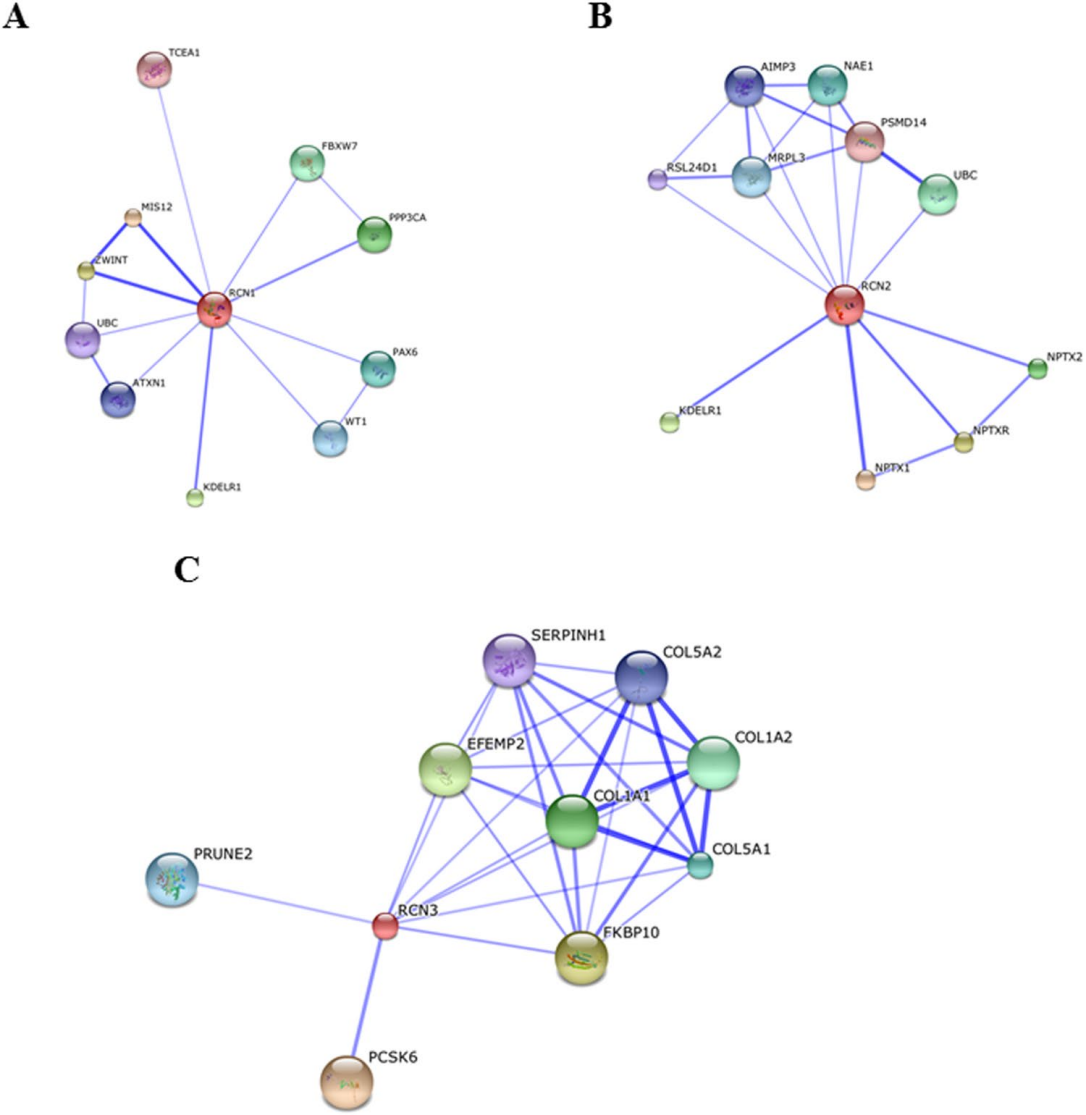
Protein interactome network for RCN proteins. Functional interactors of RCN-1 **(A)**, RCN-2 **(B)**, and RCN-3 **(C)** extracted from STRING tool. This database includes interactions from published literature describing experimentally studied interactions, as well as those from genome analysis using several well-established methods based on domain fusion, phylogenetic profiling and gene neighbourhood concepts. Accordingly, a confidence score for every protein–protein association is assigned to the network. A higher score is assigned when an association is supported by several types of evidence. To minimize false positives as well as false negatives, all interactions tagged as “low-confidence” (<0.150) in STRING database were eliminated from this study. Proteins are represented with nodes and the interactions with continuous lines represent direct interactions. Stronger associations are represented by thicker lines.

Using STRING software^14^, we performed a protein-scale interaction network of the RCN family. Protein interactome networks for RCN-1 (Fig. 2A), RCN-2 (Fig. 2B) and RCN-3 (Fig. 2C) revealed differences between RCN proteins. Interestingly, RCN-3 was specifically interconnected with collagen network (Fig. 2C). This functional association points out a direct link between RCN-3 and collagen type I expression, suggesting mechanistic clues to the potential regulatory effects between both proteins.

### Effects of RCNs on ECM components in human cardiac fibroblasts

**Figure 3:**
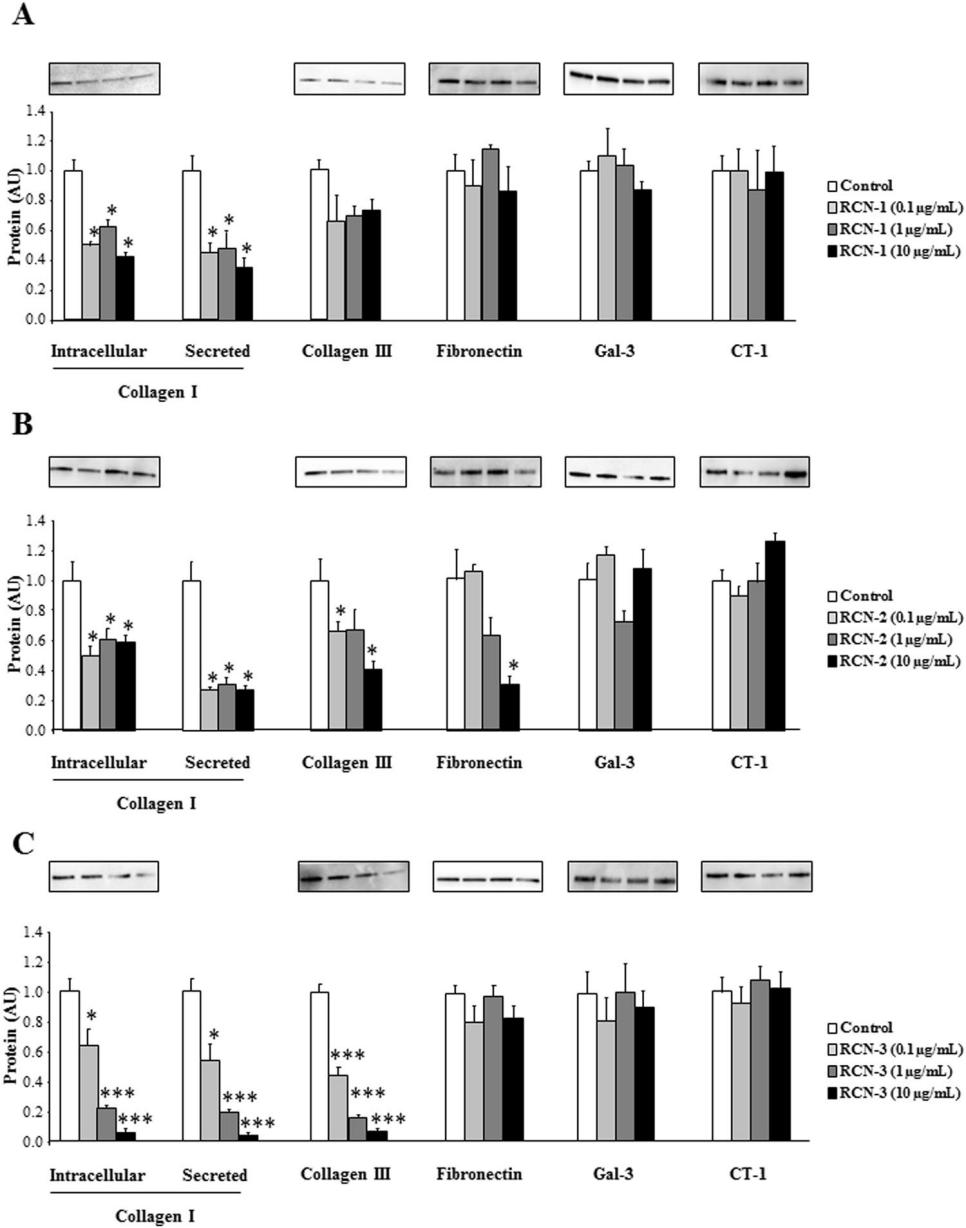
Effects of RCN family on ECM components in human cardiac fibroblasts. Dose-response (0.1, 1 and 10 µg/mL) of RCN-1 **(A)**, RCN-2 **(B)** and RCN-3 **(C)** on collagen type I, collagen type III, fibronectin, Gal-3 and CT-1 protein levels in human cardiac fibroblasts. Histogram bars represent the mean ± SEM (n > 6 wells at 70% of confluence per condition) of the three independent experiments. Densitometry values were normalized to stain free gel. *p < 0.05; **p < 0.01; ***p < 0.001 *vs*. Control.

We next analyze the effects of RCNs on ECM production in human cardiac fibroblasts. Treatment with recombinant RCN-1 decreased intracellular and secreted collagen type I protein levels at all the doses employed (Fig. 3A). In contrast, RCN-1 was not able to modify the protein expression of collagen type III, fibronectin, Gal-3 or CT-1 (Fig. 3A) with any of the doses studied.

Treatment with RCN-2 decreased intracellular and secreted collagen type I protein levels (Fig. 3B). In addition, RCN-2 was able to decreased collagen type III and fibronectin protein levels in a dose-dependent manner without modification in Gal-3 or CT-1 protein levels (Fig. 3B).

RCN-3 treatment decreased intracellular and secreted collagen type I and collagen type III protein levels in a dose-dependent manner without modifications in fibronectin, Gal-3 or CT 1 protein levels (Fig. 3C).

### Effects of RCN-3 on intracellular pathways human cardiac fibroblasts

**Figure 4:**
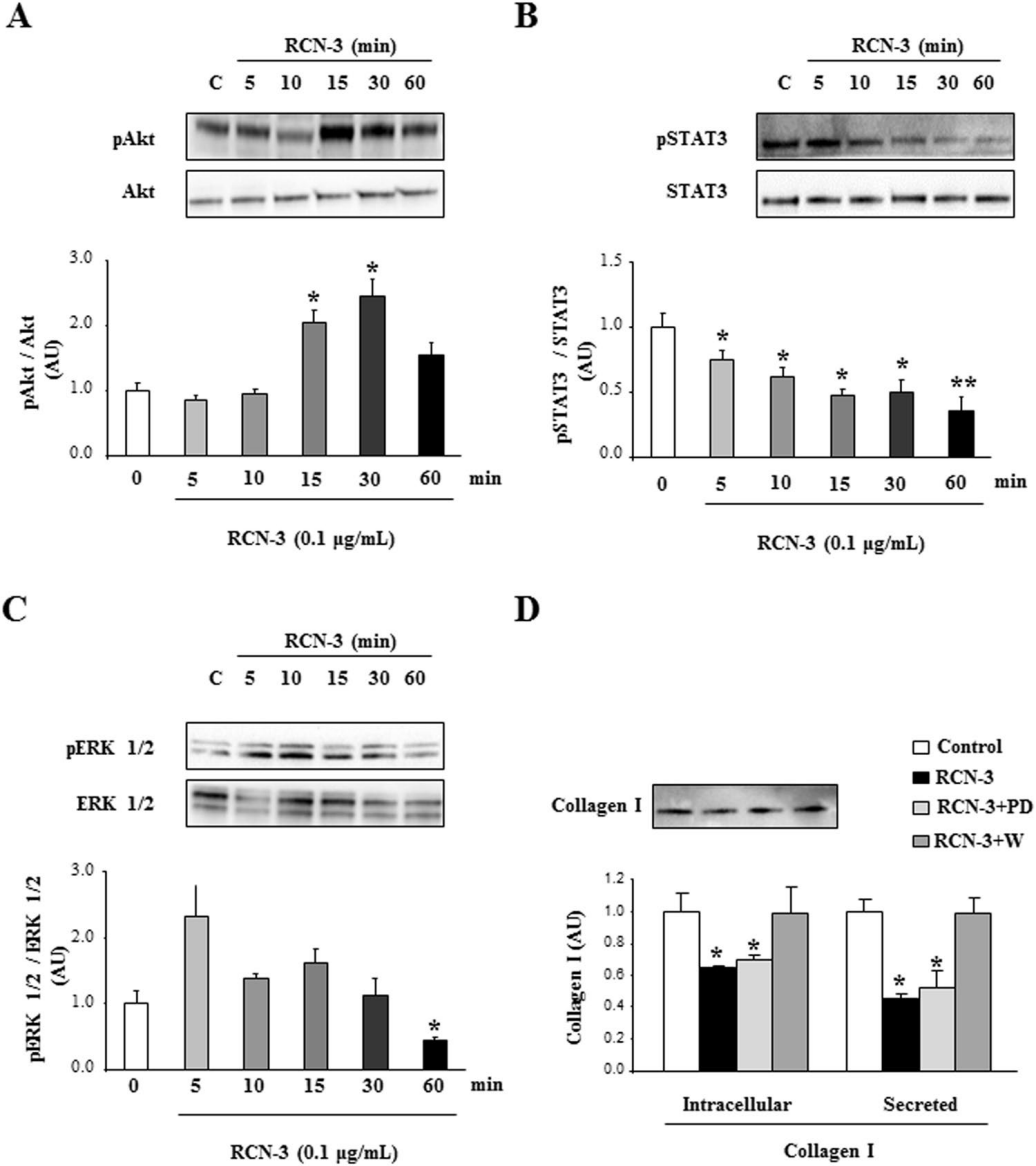
Akt mediates the antifibrotic effect of RCN-3 in human cardiac fibroblasts. Human cardiac fibroblasts were treated with RCN-3 (0.1 µg/mL) for 5, 10, 15, 30 and 60 minutes. Effects of RCN-3 (0.1 µg/mL) on Akt **(A)**, STAT3 **(B)** and ERK 1/2 **(C)** at 5, 10, 15, 30 and 60 minutes in human cardiac fibroblasts. Effect of PD98059 (10^−5^M) and Wortmannin (10^−5^M) on the decrease of collagen type I protein levels induced by RCN-3 (0.1 µg/mL) at 24 hours in human cardiac fibroblast **(D)**. Histogram bars represent the mean ± SEM (n > 6 wells at 70% of confluence per condition) of the three independent experiments. Densitometry values were normalized to stain free gel. *p < 0.05; **p < 0.01; *vs*. Control. ^†^p < 0.05 *vs*. RCN-3.

The possible intracellular mechanisms by which RCN-3 exerts its antifibrotic effects in human cardiac fibroblasts were analyzed. In human cardiac fibroblasts, recombinant RCN-3 (0.1 µg/ml) increased Akt phosphorylation after 15 and 30 minutes of stimulation (Fig. 4A) and decreased STAT3 phosphorylation in all the times studied (5–60 minutes) (Fig. 4B). In addition, recombinant RCN-3 decreased ERK 1/2 phosphorylation after 60 min of stimulation (Fig. 4C).

The presence of PD98059, a specific inhibitor of ERK 1/2, was not able to modify the decrease in collagen type I protein levels induced by RCN-3 (Fig. 4D). In contrast, the presence of Wortmannin, a specific inhibitor of Akt, prevented the decrease in intracellular and secreted collagen type I protein levels induced by RCN-3 at 24 hours of stimulation (Fig. 4D).

### Over-expression of RCN-3 decreases collagen type I protein levels in human cardiac fibroblasts

**Figure 5:**
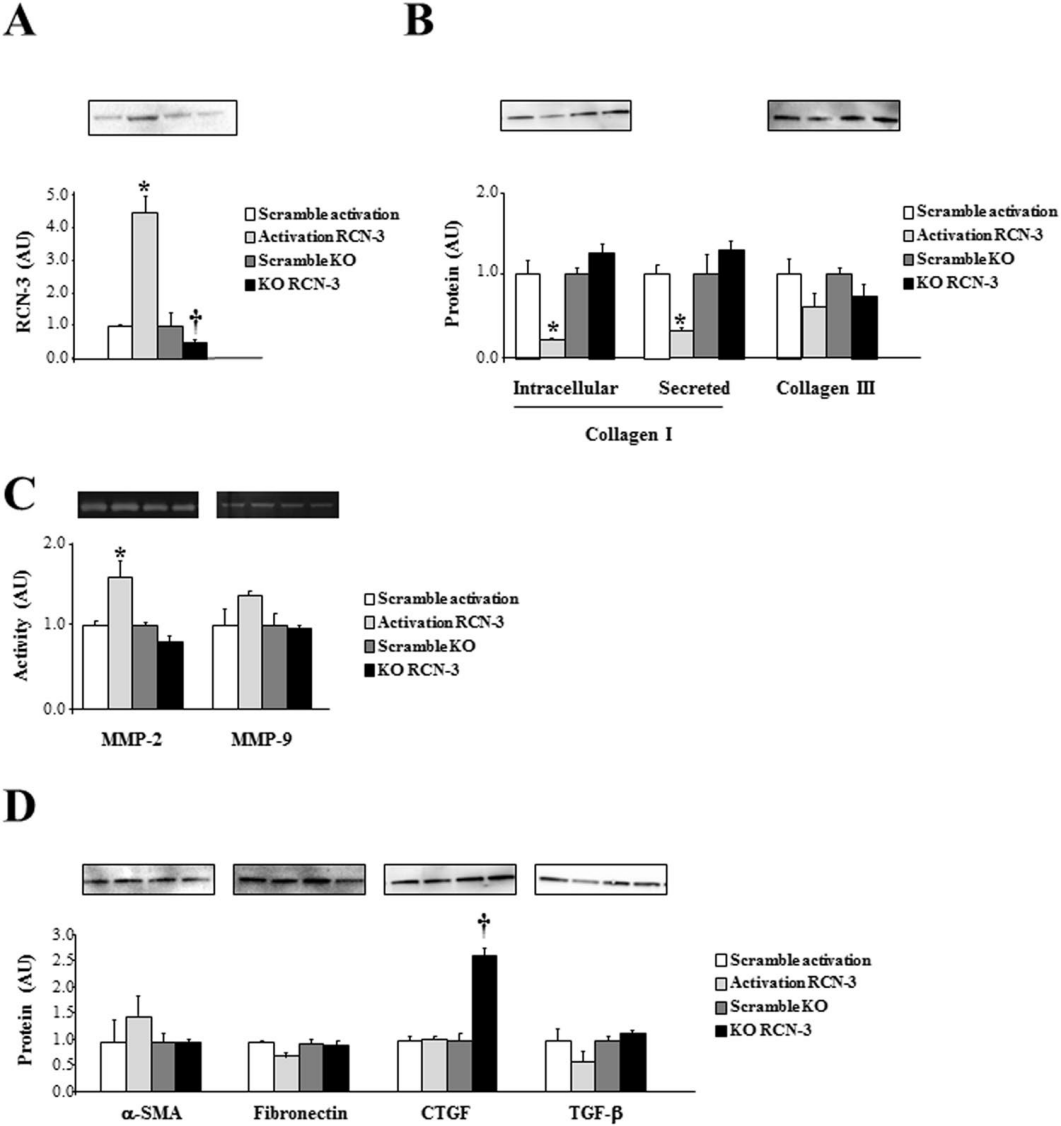
Effects of RCN-3 overexpression and knockout in human cardiac fibroblasts. RCN-3 CRISPR/Cas9 system was used in cardiac fibroblasts. RCN-3 protein expression was increased in overexpressed RCN-3 cells and decreased in RCN-3 knockout cells **(A)**. Collagen type I and collagen type III protein levels **(B)**; MMP-2 and MMP-9 activities **(C)**; α-SMA, fibronectin, CTGF and TGF-β **(D)** in over-activation and knockout RCN-3 human cardiac fibroblasts. Histogram bars represent the mean ± SEM (n > 6 wells at 70% of confluence per condition) of the three independent experiments. Densitometry values were normalized to stain free gel. *p < 0.05 *vs*. Scramble activation; ^†^p < 0.05 *vs*. Scramble knockout.

To further elucidate the role of RCN-3 in human cardiac fibroblasts, we promoted the overexpression and the knock-down of RCN-3 in these cells. We confirmed the increase or decrease in RCN-3 protein levels in cells with RCN-3 over-expression or in RCN-3-knock-down cells respectively (Fig. 5A). Over-activation of RCN-3 promoted a decrease in intracellular and secreted collagen type I protein levels in human cardiac fibroblasts at 24 hours of stimulation (Fig. 5B) without modification in collagen type III protein levels (Fig. 5B). RCN-3-knock-down cells presented the same levels of collagen type I and collagen type III protein levels as compared to control cells (Fig. 5B). RCN-3 over-activation induced an increase in metalloproteinase (MMP)-2 activity without modification in MMP-9 activity. In contrast, RCN-3 knock-down did not alter the activity of these MMPs (Fig. 5C). RCN-3 over-activation or knock-down did not modify the expression of different ECM proteins such as α-SMA, fibronectin or TGF-β (Fig. 5D), except the increase in CTGF protein levels observed in RCN-3-knock-down cells (Fig. 5D).

### RCN-3 blocks the increase in collagen type I induced by several profibrotic stimulus

**Figure 6:**
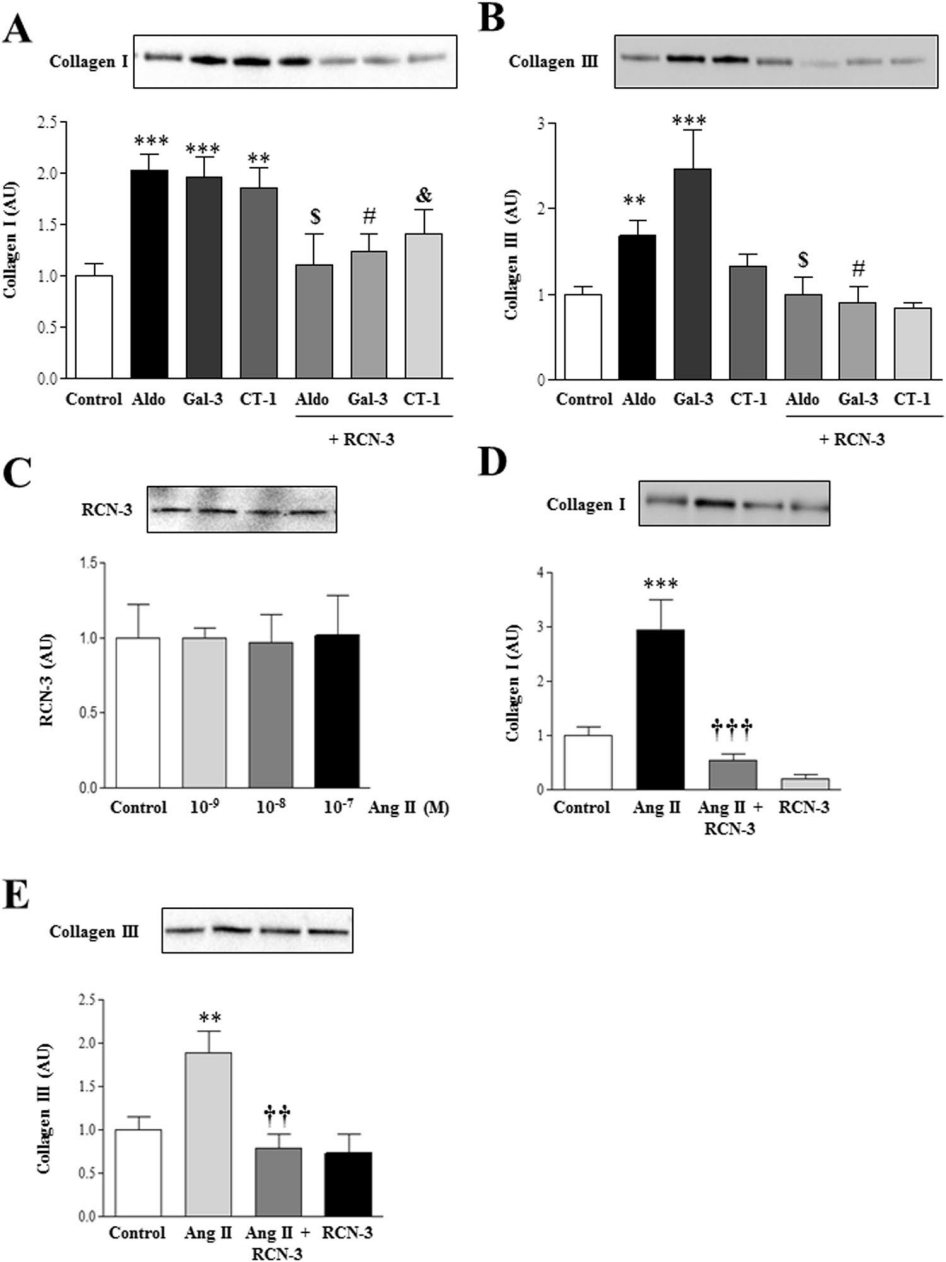
RCN-3 blocked collagen induction by several profibrotic stimulus. Effect of recombinant RCN-3 (0.1 µg/mL) on collagen type I **(A)** and collagen type III **(B)** protein levels induced by Aldo (10^−8^M, Gal-3 (10^−8^M) and CT-1 (10^−7^M) in human cardiac fibroblasts. Dose-response of angiotensin II (Ang II; 10^−9^M-10^−7^M) on RCN-3 **(C)**. Effects of RCN-3 (0.1 µg/mL) on collagen type I **(D)** and collagen type III **(E)** protein levels induced by Ang II (10^−7^M) in human cardiac fibroblasts. Histogram bars represent the mean ± SEM (n > 6 wells at 70% of confluence per condition) of the three independent experiments. Densitometry values were normalized to stain free gel. *p < 0.05; **p < 0.01; ***p < 0.001 *vs*. Control. ^$^p < 0.05 *vs*. Aldo. ^#^p < 0.05 *vs*. Gal-3. ^&^p < 0.05 *vs*. CT-1. ^††^p < 0.01; ^†††^p < 0.001 *vs*. Ang II.

The addition of recombinant RCN-3 was able to prevent the increase in collagen type I protein levels induced by Aldo, Gal-3 and CT-1 in human cardiac fibroblasts (Fig. 6A). In addition, treatment with RCN-3 prevented the enhanced collagen type III protein levels induced by Aldo and Gal-3 (Fig. 6B).

Treatment with Ang II did not modify RCN-3 expression at any of the doses employed (Fig. 6C). Ang II treatment increased collagen type I and III expressions in a dose-dependent manner (Data not shown). The addition of RCN-3 blunted the increase in collagen type I (Fig. 6D) and collagen type III (Fig. 6E) induced by Ang II in human cardiac fibroblasts.

## Discussion

HF is considered as a complex disorder in which neuro-hormonal, inflammatory and immunity systems are involved^15^. During the development of HF, cardiac fibrosis and hypertrophy occur, changing heart geometry, structural organization and gene expression^16^. These modifications play a crucial role in the genesis of arrhythmias and deterioration of both systolic and diastolic function. Despite optimal therapy, patients with HF experience clinically meaningful disease progression^17^, being necessary the discovery of new mechanisms and novel therapeutics targets for the treatment of cardiac fibrosis and hypertrophy.

The purpose of this study was to identify new proteins modulated by three related-profibrotic agents: Aldo, Gal-3 and CT-1. Using a proteomic approach we identified RCN-3 as a new target down-regulated by Aldo, Gal-3 and CT-1. In addition, recombinant RCN-3 exerted antifibrotic effects in human cardiac fibroblasts and was able to block the profibrotic effect of Aldo, Gal-3, CT-1 and Ang II in cardiac fibroblasts. Thus, RCN-3 emerges as a negative modulator of collagen regulation in human cardiac fibroblasts and as a potential new mediator of cardiac fibrosis.

RCNs can be classified into the EF-hand calcium-binding protein superfamily, which includes calmodulin, troponin C, and myosin light chain^18^. The function of RCN proteins remains unknown. However, its localization in the lumen of the endoplasmic reticulum suggests a role in protein synthesis, modification, and intracellular transport. Dysregulation of RCN proteins has been reported in various diseases, such as cancer, cardiovascular, and neuromuscular diseases^19–21^. In the present study we found that Aldo, Gal-3 and CT-1 decreased RCN-3 protein levels in human cardiac fibroblasts. RCN-3 mRNA levels were not modified under any of the treatments at 24 hours (Supplementary Figure S1). Thus, we can conclude that RCN-3 decrease could be due to protein degradation. However, new studies need to analyze more in depth the mechanisms underlying RCN-3 degradation. The decrease in RCN-3 protein levels induced by Aldo is dependent on MR activation since the presence of Spirolonactone, a MR antagonist, was able to block the decrease in RCN-3 protein levels induced by Aldo in human cardiac fibroblasts. In previous studies from our group, we have demonstrated that the employment of a MR antagonist was able to block Aldo-induced CT-1 and Gal-3 expressions^6,7,22^, suggesting an important role of MR in RCN-3 expression. All the isoforms of RCN decreased collagen type I protein levels in basal conditions, although RCN-3 promoted the most important decrease in collagen production and secretion. Interestingly, it has been published that down-regulation of RCN-1 leads to a hypertrophic phenotype in cardiomyocytes, RCN-1 being a negative modulator of cardiomyocyte hypertrophy^23^. Altogether, RCNs could emerge as key modulators of the hypertrophic and fibrotic response under pathological conditions. The differences observed between RCN-1, RCN-2 and RCN-3 could be explained by the fact that RCN-3 shows only 55 and 36% homology with RCN-1 and RCN-2 respectively, suggesting that RCN-3 could have a different function than the others members of the RCN family^24^.

There is few information concerning RCN-3 and its effects. RCN-3 is ubiquitously expressed, but its role is still unknown. It should play an important role in the maintenance of normal cell behavior because the homozygous mutation in mice causes neonatal lethality^24^. RCN-3 is upregulated in GH4C1 cells and interacts with proPACE4, a member of subtilisin-like proprotein convertase family, stimulating its activation and secretion^18^. It is also upregulated in endothelial cells activated by multiple sclerosis serum^25^. In the present study, we show for the first time that recombinant RCN-3 is able to prevent the profibrotic effects of different molecules such as Aldo, Gal-3 and CT-1. We also studied the possible role of different intracellular pathways involved in cardiac fibrosis^26–29^. RCN-3 modified the phosphorylation of Akt, STAT3 and ERK 1/2. Our data show that Akt pathway mediates the antifibrotic effects of RCN-3 since only the presence of the Akt inhibitor was able to prevent the decrease in intracellular and secreted collagen I protein levels induced by RCN-3. We extended its effects to another profibrotic agent, Ang II. In human cardiac fibroblasts, Ang II enhanced collagen levels, effect that was blocked by RCN-3 co-treatment, confirming its role as antifibrotic agent. In parallel, RCN-1 blocked cardiomyocyte hypertrophy induced by phenylephrine^23^. Altogether these data suggest the beneficial effects of RCN molecules in both cardiac fibrosis and hypertrophy.

In summary, we identified RCN-3 as a novel protein down-regulated by Aldo, Gal-3 and CT-1. RCN-3 decreased collagen production and inhibited agonist induced collagen expression. Therefore, to enhance RCN-3 levels may represent a new therapeutic option in the treatment of cardiac pathologies in which cardiac fibrosis is involved.

## Methods

### Cell culture

Human Cardiac Fibroblasts were obtained from Promocell and maintained in medium Fibroblasts Media 3. Cells were cultured according to the manufacturer´s instructions. Cells were used between passages 5–7. Cells were seeded into six-well plates at 90% confluence and serum starved for 12 h and then stimulated with Aldosterone (10^−8^ M, Sigma), Gal-3 (10^−8^ M, R&D Systems), CT-1 (10^−7^ M, R&D Systems), RCN-1 (0.01, 0.1 and 10 µg/mL, Abcam), RCN-2 (0.01, 0.1 and 10 µg/mL, Abcam), RCN-3 (0.01, 0.1 and 10 µg/mL, Abcam), Spironolactone (Spiro, 10^−6^M, Sigma) and Angiotensin II (Ang II, 10^−9^–10^−7^M, Sigma) for 24 hours for protein analysis. Aldosterone was prepared in ethanol at 10^−2^M and then diluted in culture medium to be used at 10^−8^M. Recombinant Gal-3 and CT-1 were reconstituted in PBS. The doses were chosen based on preliminary and previous studies^6,22,30^.

For the intracellular pathways study, cells were treated with RCN-3 for 5, 15, 30 and 60 minutes. The following chemical inhibitors were added at 10^−5^ mol/L 1 hour prior to RCN-3 stimulation: Wortmannin (Sigma Aldrich), PD98059 (Sigma Aldrich) and AG-490 (Sigma Aldrich).

### Mass spectrometry based-quantitative proteomics

A shotgun comparative proteomic analysis of untreated cardiac fibroblasts and cardiac fibroblasts stimulated with Aldo, Gal-3, or CT-1 was performed using iTRAQ (isobaric Tags for Relative and Absolute Quantitation)^31^ (See supplementary information for technical details). Global experiments were carried out with three biological replicates in each experimental condition. Peptide labeling, peptide fractionation, and mass-spectrometry analysis, were performed as previously described^32^. After MS/MS analysis, protein identification and relative quantification were performed with the ProteinPilot™ software (version 4.5; Sciex) using the Paragon™ algorithm as the search engine^33^. Although relative quantification and statistical analysis were provided by the ProteinPilot software, an additional 1.3-fold change cutoff for all iTRAQ ratios (ratio < 0.77 or > 1.3) and a p-value lower than 0.05 were selected to classify proteins as up- or down-regulated (at least in two of three biological replicates). Proteins with iTRAQ ratios below the low range (0.77) were considered to be under-expressed, whereas those above the high range (1.3) were considered to be overexpressed.

### CRISPR/Cas9 genome editing mediated deletion/activation of RCN-3

The knock-down and the activation of RCN-3 in human cardiac fibroblasts was performed by CRISPR/Cas9 (clustered regularly interspaced short palindrome repeats) guided genome editing. Cells were seeded into 6-well plates at 70% confluence and transfected with a pool of three plasmids, each encoding the Cas9 nuclease and a target-specific 20 nt guide RNA designed for maximum knockout/activation efficiency according to the manufacturer’s instructions (Santa Cruz Biotechnology). Scramble gRNA CRISPR/Cas9 Plasmid were used as a control.

### Western Blot

Total proteins from human cardiac fibroblast were separated by SDS-PAGED on 10% polyacrylamide gels and transferred to Hybond-c Extra nitrocellulose membranes (Amersham Biosciences, Piscataway, NJ). Membranes were probed with primary antibodies for RCN-1 (Abcam; dilution 1:500), RCN-2 (Abcam; dilution 1:500), RCN-3 (Abcam; dilution 1:500), collagen I (Sigma; dilution 1:500), collagen III (Santa Cruz; dilution 1:500), Fibronectin (Millipore; dilution 1:1000), Gal-3 (ThermoFisher; dilution 1:1000), CT-1 (Abcam; dilution 1:500), connective tissue growth factor (CTGF; Torrey Pines Biolabs Inc., dilution 1/1000), ERK1/2 and ERK1/2-P (Thr202/Tyr204) at 1/1000 (Cell Signaling), Akt and Akt-P (Ser473) at 1/1500 (Cell Signaling), Stat3 and Stat3-P (Tyr705) at 1/1500 (Cell Signaling). Western blots were performed with stain-free gels for loading control. After washing, detection was made through incubation with peroxidase-conjugated secondary antibody, and developed using an ECL chemiluminescence kit (Amersham). After densitometric analyses, optical density values were expressed as arbitrary units. Results are expressed as an n-fold increase over the values of the control group in densitometric arbitrary units. All Western Blots were performed at least in triplicate for each experimental condition.

### ELISA

Collagen type I was measured in the supernatants of the cells by ELISA according to the manufacturer’s instructions (R&D Systems).

### Gelatin zymography

Aliquots of culture media containing 25 μl of supernatant were resolved on a 10% SDS polyacrylamide gel containing 0.3% gelatin. The gel was rinsed three times for 15 min with a solution of 2.5% Triton × 100 to remove SDS and renature the proteins, followed by incubation for 48 h at 37 °C in 1000 mmol/l Tris-HCl, pH 7.5 with 1000 mmol/l CaCl_2_ and 5000 mmol/l NaCl to promote degradation of gelatin. Gels were fixed in 40% methanol and 10% acetic acid, and then stained for 30 min in 0.25% Coomassie blue R-250 to identify proteolytic activity of MMPs.

### Statistical analyses

Data are expressed as mean ± SEM. Normality of distributions was verified by means of the Kolmogorov–Smirnov test. Data were analyzed using a one-way analysis of variance, followed by a Newman-Keuls to assess specific differences among groups or conditions using GraphPad Software Inc. Pearson correlation coefficients were calculated to determine correlations. The predetermined significance level was P < 0.05.

## Electronic supplementary material

Supplementary data
