# Supplementary material for: Differential Proteomics Identifies Reticulocalbin-3 as a Novel Negative Mediator of Collagen Production in Human Cardiac Fibroblasts
Source: Sci Rep. 2017 Sep 22;7:12192. doi: 10.1038/s41598-017-12305-7 (PMC5610303; doi:10.1038/s41598-017-12305-7)
Supplement: Supplementary file 1 — Supplementary data [file 41598_2017_12305_MOESM1_ESM.pdf]

DIFFERENTIAL PROTEOMICS IDENTIFIES RETICULOCALBIN-3 AS A NOVEL NEGATIVE MEDIATOR OF COLLAGEN PRODUCTION IN HUMAN CARDIAC FIBROBLASTS

Ernesto Martínez-Martínez[1,2], Jaime Ibarrola[1], Amaya Fernández-Celis[1], Enrique Santamaria[3], Joaquín Fernández-Irigoyen[3], Patrick Rossignol[4], Frederic Jaisser[2,4], Natalia López-Andrés*[1,4]

[1] Cardiovascular Translational Research. Navarrabiomed (Fundación Miguel Servet), Instituto de Investigación Sanitaria de Navarra (IdiSNA), Pamplona. Spain; [2]INSERM UMRS 1138 Team 1, Centre de Recherche des Cordeliers, University Pierre and Marie Curie, Paris, France ; [3] Proteored-ISCIII, Proteomics Unit, Navarrabiomed, Departamento de Salud, Universidad Pública de Navarra, IDISNA, Navarra Institute for Health Research, Pamplona, Spain; [4] INSERM, Centre d'Investigations Cliniques-Plurithématique 1433, UMR 1116 Université de Lorraine, CHRU de Nancy, FCRIN INI-CRCT, France

***Corresponding Author:** Natalia López-Andrés. Cardiovascular Translational Research. Navarrabiomed (Miguel Servet Foundation). C/Irunlarrea 3, 31008 Pamplona, Spain. Telephone: 34-848422359. Fax: 34-848422300 E-mail: natalia.lopez.andres@navarra.es

Supplementary methods

Mass spectrometry based-quantitative proteomics

Sample preparation and peptide labeling. Cellular pellets derived from untreated cardiac fibroblasts and stimulated cardiac fibroblasts were resuspended in lysis buffer containing 7 M urea, 2 M thiourea, 4% (v/v) CHAPS, 50 mM DTT. Homogenates were spinned down at 14,000 x rpm for 1 h at 15ºC. Protein concentration was measured in the supernatants with the Bradford assay kit (Bio-rad). Protein extracts (160 µg) were precipitated with methanol/choloroform, and pellets dissolved in 7M urea, 2 M thiourea, 4% (v/v) CHAPS. Protein quantitation was performed with the Bradford assay kit (Bio-Rad). iTRAQ labeling of each sample was performed according to the manufacturer's protocol (Sciex). Briefly, a total of 80 µg of protein from each cellular condition was reduced with 50 mM tris (2-carboxyethyl) phosphine (TCEP) at 60 °C for 1 h, and cysteine residues were alkylated with 200 mM methylmethanethiosulfonate (MMTS) at room temperature for 15 min. Protein enzymatic cleavage was carried out with trypsin (Promega; 1:20, w/w) at 37 °C for 16 h. Three independent iTRAQ experiments were performed: i) untreated cardiac fibroblasts vs Aldo-stimulated cardiac fibroblasts, ii) untreated cardiac fibroblasts vs CT-1-stimulated cardiac fibroblasts, and iii) untreated cardiac fibroblasts vs Gal-3-stimulated cardiac fibroblasts. Each tryptic digest derived from each biological replicate was labelled according to the manufacturer's instructions with one isobaric amine-reactive tags. After 1h incubation, each set of labelled samples were independently pooled and evaporated until < 40 µl in a vacuum centrifuge. To increase the proteome coverage, the peptide pool was injected to an Ettan LC system with a X-Terra RP18 pre-column (2.1 x 20mm) and a high pH stable X-Terra RP18 column (C18; 2.1 mm x 150mm; 3.5µm) (Waters) at a flow rate of 40 µl/min. Peptides were eluted with a mobile phase B of 5–65% linear gradient over 35 min (A, 5 mM

ammonium bicarbonate in water at pH 9.8; B, 5 mM ammonium bicarbonate in acetonitrile at pH 9.8). For each iTRAQ experiment, fractions were collected, evaporated under vacuum and reconstituted into 20 µl of 2% acetonitrile, 0.1% formic acid, 98% MilliQ-H20 prior to mass spectrometric analysis.

Mass Spectrometry Analysis. Peptides mixtures were separated by reverse phase chromatography using an Eksigent nanoLC ultra 2D pump fitted with a 75 µm ID column (Eksigent 0.075 x 150). Samples were first loaded for desalting and concentration into a 0.5 cm length 300 µm ID pre-column packed with the same chemistry as the separating column. Mobile phases were 100% water 0.1% formic acid (FA) (buffer A) and 100% Acetonitrile 0.1% FA (buffer B). Column gradient was developed in a 70 min two step gradient from 2% B to 30% B in 60 min and 30%B to 40% B in 10 min. Column was equilibrated in 95% B for 5 min and 2% B for 15 min. During all process, pre-column was in line with column and flow maintained all along the gradient at 300 nl/min. Eluting peptides from the column were analyzed using an AB Sciex 5600 TripleTOF™ system. Information data acquisition was acquired upon a survey scan performed in a mass range from 350 m/z up to 1250 m/z in a scan time of 250 ms. Top 25 peaks were selected for fragmentation. Minimum accumulation time for MS/MS was set to 75 ms giving a total cycle time of 2.1 s. Product ions were scanned in a mass range from 100 m/z up to 1700 m/z and excluded for further fragmentation during 15 s. After MS/MS analysis, data files were processed using ProteinPilot™ 4.5 software from AB Sciex which uses the algorithm Paragon™ (v.4.0.0.0)[1] for database search and Progroup™ for data grouping and searched against Uniprot human database. False discovery rate was performed using a non-lineal fitting method and displayed results were those reporting a 1% Global False Discovery Rate (FDR) or better. Relative quantification and protein identification were performed with the

ProteinPilot™ software (version 4.5; Sciex) using the Paragon™ algorithm as the search engine. Each MS/MS spectrum was searched against a database of human protein sequences (Uniprot). The search parameters allowed for cysteine modification by MMTS and biological modifications programm in the algorithm (i.e. phosphorylations, amidations, semitryptic fragments, etc.). Reporter ion intensities were bias corrected for the overlapping isotope contributions from the iTRAQ tags according to the certificate of analysis provided by the reagent manufacturer (Sciex). The peptide and protein selection criteria for relative quantitation were performed as follows. Only peptides unique for a given protein were considered for relative quantitation, excluding those common to other isoforms or proteins of the same family. Proteins were identified on the basis of having at least one peptide with an ion score above 99% confidence. Among the identified peptides, some of them were excluded from the quantitative analysis for one of the following reasons: (i) The peaks corresponding to the iTRAQ labels were not detected; (ii) the peptides were identified with low identification confidence (<1.0%); (iii) the sum of the signal-to-noise ratio for all of the peak pairs was <6 for the peptide ratios. The protein sequence coverage (95% conf.) was estimated for specific proteins by the percentage of matching amino acids from the identified peptides having confidence greater than or equal to 95% divided by the total number of amino acids in the sequence. Several quantitative estimates provided for each protein by ProteinPilot were utilized: the fold change ratios of differential expression between labelled protein extracts; the p-value, representing the probability that the observed ratio is different than 1 by chance. A decoy database search strategy was also used to estimate the false discovery rate (FDR), defined as the percentage of decoy proteins identified against the total protein identification. The FDR was calculated by searching the spectra against the decoy database generated from the target database. The results

were then exported into Excel for manual data interpretation. Although relative quantification and statistical analysis were provided by the ProteinPilot software, an additional 1.3-fold change cutoff for all iTRAQ ratios (ratio <0.77 or >1.3) and a p-value lower tan 0.05 were selected to classify proteins as up- or down-regulated (at least in two of three biological replicates). Proteins with iTRAQ ratios below the low range (0.77) were considered to be underexpressed, whereas those above the high range (1.3) were considered to be overexpressed.

References

1. Shilov IV, Seymour SL, Patel AA, Loboda A, Tang WH, Keating SP, Hunter CL, Nuwaysir LM, Schaeffer DA. The Paragon Algorithm, a next generation search engine that uses sequence temperature values and feature probabilities to identify peptides from tandem mass spectra. *Mol Cell Proteomics* 2007;**6**:1638-1655.

SUPPLEMENTAL TABLES

Supplementary table 1. Galectin-3- and cardiotrophin-1-modulated reticulocalbin family proteins.

**Protein Name**	**Gene name**	**Uniprot code**	**Ratio Con2: Con1**	**P-value Con2:Con1**	**Ratio Gal1:Con1**	**Ratio Gal2:Con1**	**Ratio Gal3:Con1**	**P-value Gal 1:Con1**	**P-value Gal 2:Con1**	**P-value Gal 3:Con1**
Down-regulation in Gal-3-treated cells										
Reticulocalbin-1	RCN1	Q15293	0.97	0.71	0.60	0.59	0.45	0.03	0.06	0.01
Reticulocalbin-3	RCN3	Q96D15	0.81	0.13	0.57	0.59	0.51	0.04	0.05	0.01
	**Gene name**	**Uniprot code**	**Ratio Con2: Con1**	**P-value Con2:Con1**	**Ratio CT1:Con1**	**Ratio CT2:Con1**	**Ratio CT3:Con1**	**P-value CT 1:Con1**	**P-value CT2:Con1**	**P-value CT 3:Con1**
Tendency to down-regulation in CT-1-treated cells										
Isoform 2 of Reticulocalbin-2	RCN2	Q14257-2	0.97	0.85	0.74	0.60	0.65	0.05	0.04	0.09

SUPPLEMENTAL FIGURES

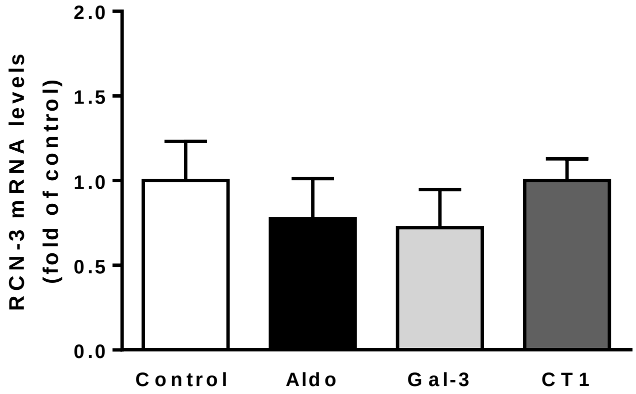

Figure S1: Effects of Aldo (10^{-8}M), Gal-3 (10^{-8}M) and CT-1 (10^{-7}M) on RCN-3 mRNA levels at 24 hours of stimulation. Histogram bars represent the mean ± SEM (n>6 wells at 70% of confluence per condition) of the three independent experiments.
